# Report on Bovine Tuberculosis

**Published:** 1897-07

**Authors:** Leonard Pearson


### BOOK REVIEWS.

REPORT ON BOVINE TUBERCULOSIS. By DR. M. H. REYNOLDS. Bulletin 51, Agricultural Experiment Station, University of Minnesota, December, 1896.

A GREAT many reports have recently been issued by Experiment Stations of this country upon the subject of bovine tuberculosis. As the experiment stations are organized, conducted for and to a large extent by the agriculturists of the country, and directly controlled by agricultural interests, it is fair to assume that there is a widespread demand for information in regard to tuberculosis, or these reports would not appear in such numbers. Most of the bulletins on this subject review with more or less thoroughness our existing knowledge in regard to tuberculosis and compile summaries and rearrange from other reports and papers in such a manner as to furnish in readily assimilable form the information that it is thought the farmers are most in need of. Therefore, there is a vast amount of repetition in them, and if one undertakes to read each bulletin on tuberculosis as it appears the task becomes exceedingly monotonous. The bulletin recently issued in Minnesota presents much of this old subject in a new way; it makes new applications of existing knowledge, and brings out some new facts; therefore Dr. Reynolds's work is very refreshing. The publication covers about seventy pages, and is illustrated with eight full-page half-tone plates.

It begins with a summary of its contents in which the principal points of the subject-matter are expressed in a few short, pithy sentences or paragraphs. The whole subject is treated in a conservative, scientific spirit that tends to inspire confidence, and sweeping statements in reference to undemonstrated points seem to be studiously avoided. The following sentence strikes a keynote, it will bear frequent repetition, and covers points in regard to which there has been much misapprehension: "Sanitarians are not quarrelling with butter, milk, or meat—not with dairymen nor breeders, but wholly with tuberculosis. We cannot well do without milk or butter, nor do we wish to do without meat; but let us have them pure and wholesome. No man can do a better thing for his neighbor than to show him truth. There is safety when we see, and danger when we do not."

The absurd charges that have been made against the use of tuberculin from time to time are effectually disposed of. The matter of meat-, milk-, and dairy-inspection is considered, and the laws of Minnesota, together with the ordinances and regulations operating in Minneapolis, and the court decisions bearing on these statutes, are given in full. Reference is made to the laws regarding tuberculosis of cattle in operation in Switzerland, Denmark, and France, and then follows a report upon the effect of tuberculin upon non-tuberculous cows and upon fattening steers, in which it is shown that tuberculin does not injure a healthy animal, so that previous results of this kind receive new confirmation.

The resolutions on tuberculosis adopted by the United States Veterinary Medical Association at its meetings in 1895 and in 1896 are given in full. A significant table shows the percentage of tuberculosis that has been found in herds of different breeds kept under different conditions. It tends to show that there is a distinct relation between unsanitary conditions and the prevalence of tuberculosis. Another table shows the location of tuberculous lesions in a number of animals examined post-mortem, and still another table gives the temperature reactions. Statistics of this character are all too few, and every contribution of this sort is of great value to the student of tuberculosis. It is only by the accumulation and careful digestion of large masses of material of this kind that reliable conclusions can be drawn. Some work is done upon the lines first laid down by Professor Bang, and it is demonstrated again that healthy calves can be raised from tuberculous cows, provided certain precautions are observed and that under some circumstances it is profitable to retain reacting animals; but the point is made very clearly that it is a costly experiment to keep animals, tuberculous in any degree, in a herd that is otherwise healthy, and where such animals are retained they must be isolated carefully. A conservative view is taken as to what can best be done to suppress tuberculosis, and general sanitary measures are recommended, most of which is summed up in the following paragraph: "Sanitarians should insist that Nature's laws are unchangeable, and that the great laws of physiology and heredity will never be varied so that breeders may acquire fancy muzzles for cattle or belts of color around the body. The man who is founding a herd should be very sure that he is not founding a tuberculous herd. He should try to breed cattle, not tuberculosis. The great value of the tuberculin-test to breeders lies in the fact that it enables them to know whether their cattle are free from tuberculosis, and it enables them to free their herds and put them on sound and healthy bases in case they are diseased."

Some attention is given to the curative value of tuberculin, and it is found that of eleven animals treated two made evident recoveries and two showed recent repair in diseased tissues.

Of course, the *Bulletin* is prepared principally for farmers, and technical readers are asked to remember that it has been necessary to employ throughout such language as would make it intelligible for stockmen. There are a few statements, however, that all veterinarians will not be quite willing to accept without further data; for instance, it is stated that, "there is no ground for argument concerning the use of meat and milk from tuberculous cattle." Is it not true that this statement requires some modification? While there are very few who care to use the milk of any cow that has responded to the tuberculin-test, unless it were heated to a point that would insure the destruction of the tubercle bacillus, there are many who have given careful attention to this subject who do not feel that there is any risk in using the flesh of animals in certain stages of disease, and in the foreign countries in which meat-inspection is most developed such flesh is constantly placed upon the market without evil results so far as can be ascertained. It is also stated that the place to examine a sample of meat or milk for soundness in respect to tuberculosis is the stable, and the only method at present the tuberculin-test. Meat-inspectors who are accustomed to make their examinations upon the floor of the slaughter-house will hardly agree with the first part of this statement, but still, we must remember that where there is an ineffectual attempt at inspection and a system that is incomplete, it is better to err by giving the consumer the benefit of the doubt than by allowing products to go on to the market that might produce disease, and fine distinctions can only be made where methods of inspection are well developed and where there is an inspecting corps large enough to enforce them. The method of making the tuberculin-test also seems a little involved and to include some unnecessary labor and time, but it is careful and accurate. The report as a whole is most excellent. It is, perhaps, the best *Bulletin* on this subject that any experiment station has published, and is calculated to be of much benefit to the live-stock interests of Minnesota.

LEONARD PEARSON.

